# Efficacy of the Fumigants Propylene Oxide and Ethyl Formate to Control Two Pest Species of Dry-Cured Hams

**DOI:** 10.3390/insects14060511

**Published:** 2023-06-01

**Authors:** Jacqueline M. Maille, M. Wes Schilling, Thomas W. Phillips

**Affiliations:** 1 Department of Entomology, Kansas State University, 123 W. Waters Hall, Manhattan, KS 66506, USA; 2 Department of Food Science, Nutrition and Health Promotion, Box 9800 Room 203 Bost Extension Center, Starkville, MS 39762, USA; schilling@foodscience.msstate.edu

**Keywords:** mold mite, ham mite, cheese mite, *Tyrophagus putrescentiae*, red-legged ham beetle, *Necrobia rufipes*, fumigation, stored products

## Abstract

**Simple Summary:**

The ham mite *Tyrophagus putrescentiae* and the red-legged ham beetle *Necrobia rufipes* are pests of dry-cured country hams that are aged for up to two years. These pests had been effectively controlled by the fumigant gas methyl bromide, which is now banned from all uses on postharvest foods except for quarantine applications. The alternative fumigants propylene oxide and ethyl formate can control several stored-product pest species but have not been studied for these ham pests. Here, we have evaluated propylene oxide and ethyl formate for controlling the ham mite and the red-legged ham beetle. Laboratory experiments of each fumigant applied to mixed life stage populations of both pests confirmed that they could be controlled at reasonable gas concentrations either with or without the presence of dry-cured ham, dog food kibbles, or fish meal in small fumigation chambers. Mite eggs were the most difficult to control. Both fumigants were effective at suppressing the mites and red-legged ham beetles at appropriate gas concentrations. While there were no negative effects of any gas being released from treated foods after fumigants were vented, these effects from the presence of commodities requires additional research. Overall, the use of propylene oxide or ethyl formate to control pests on dry-cured hams during the aging process is potentially feasible and should be studied further.

**Abstract:**

The ham mite *Tyrophagus putrescentiae* and the red-legged ham beetle *Necrobia rufipes* are harmful pests to several high-valued stored products. The regulatory phase-out of the fumigant methyl bromide necessitates the search for alternative fumigants. Propylene oxide (PPO) and ethyl formate (EF) were therefore evaluated in the laboratory for controlling these pests of dry-cured hams. Concentration–mortality studies at 25 °C of PPO and EF found that the mobile stages of the mites were very susceptible to low concentrations of 10 mg/L and less of each gas, while mite eggs were very tolerant and required 20 mg/L for PPO and 80 mg/L of EF for 100% mortality. Mixed life stage cultures of mites and beetles were treated for 24 h with either PPO or EF at 1× and 2× the estimated 99% lethal doses and confirmed effectiveness for controlling simulated pest populations. The sorptive properties of each gas in chambers with ham pieces, dog food kibbles, or fish meal were minimal for a reduction in mite toxicity when compared to treatments in empty chambers. There was no evidence that any desorbed gas occurred at a level toxic to mite eggs in any of the fumigated commodities. These fumigation studies with ham pests support further work with PPO and EF on any changes in the sensory quality of dry-cured hams for human taste and for commercial-scale fumigations toward regulatory approval.

## 1. Introduction

*Tyrophagus putrescentiae* (Schrank) (Sarcoptiformes: Acaridae) and *Necrobia rufipes* (DeGeer) (Coleoptera: Cleridae), known as the mold mite and the red-legged ham beetle, are stored-product pests associated with over 140 [1] and 120 [2] commodities, respectively. Both pests infest high-value durable products, usually containing high protein, high fat, and high moisture contents of 15–40%, such as dry-cured meats, artisanal cheeses, commercial pet food, dried fruits, and dried fish [3,4,5,6,7]. Among the pests associated with dry-cured hams, *T. putrescentiae* is considered the most important and difficult to control [8].

The control of *T. putrescentiae* and *N. rufipes* in the USA has historically been achieved via the fumigation of ham-aging structures with methyl bromide, CH_3_Br [8]. Methyl bromide is an atmospheric-ozone-depleting chemical that was recently phased out from applications to foods in many countries under the Montreal Protocol; see https://www.unep.org/ozonaction/who-we-are/about-montreal-protocol (accessed on 31 May 2023) and [8,9]. Research on methyl bromide alternatives for country ham has found that hydrogen phosphine is very effective at killing all life stages of ham mites and red-legged ham beetles [10], but related work has confirmed that the application of phosphine in ham-aging facilities has caused the severe corrosion of all electrical appliances and utilities in such buildings [8,11]. Another approved fumigant tested for ham pests was sulfuryl fluoride, but specific research for efficacy against ham mites has found that the eggs could not be killed at acceptable levels, even at triple the highest approved application rate [12]. Alternative fumigants are therefore still needed for integrated pest management programs targeting *T. putrescentiae* and *N. rufipes* to reduce economic loss to high-valued stored commodities.

Potential alternative fumigants, such as propylene oxide (C_3_H_6_O; PPO) and ethyl formate (C_3_H_6_O_2_, EF), have some approval in the USA for residues in some foods and as safe additives in personal cosmetics, but they have not been registered for use as large-scale commodity fumigants [13]. Propylene oxide is a colorless liquid at room temperature with a boiling point of 34 °C and is flammable at 3–37% volume by volume (*v*/*v*) in air [14]. The flammability of this compound is often tempered with carbon dioxide which makes it safe to use as a microbial sterilant [13,14]. PPO has been used as a microbial sterilant since 1958, and it was allowed in the USA for use as an insecticide from 1958 to 1988 when its re-registration under the amended Federal Insecticide, Fungicide and Rodenticide Act lapsed [14,15]. EF is also a colorless liquid at room temperature with a boiling point of 54 °C and is flammable at 2.8–16.5% (*v*/*v*) in air [15]. In the USA, EF is labeled as a “generally recognized as safe” (GRAS) compound and food additive by the Food and Drug Administration, with an allowance of 0.01–0.05% depending on the food item in which it is used [16]. In Australia, a commercial formulation of EF was approved as a fumigant for the disinfestation of postharvest pests in grains and other durable commodities [17]. Both “liquid fumigants” have insecticidal properties that can effectively control many species of stored-product pests, as has been reported in several studies [14,18,19,20,21,22,23,24,25].

Despite research showing that both PPO and EF can control stored-product pest species, we have not found studies on the efficacy of these methyl bromide alternatives for controlling the ham pests *T. putrescentiae* and *N. rufipes*. Therefore, the main objective of this study was to determine the efficacy of PPO and EF at controlling *T. putrescentiae* and *N. rufipes* by determining the concentration and exposure time at which the most tolerant life stages would reach 100% mortality, and to verify these findings through mixed life stage fumigations. Additionally, bioassays were used to determine if the sorption of these fumigants by various commodities could affect their efficacy against these pests, and if any desorption of the fumigants from the treated commodities occurs to any reasonable extent.

## 2. Materials and Methods

### 2.1. Ham Pest Cultures

*Tyrophagus putrescentiae* and *N. rufipes* laboratory cultures have been maintained in the Department of Entomology at Kansas State University since 2008 and were derived from active field infestations. Mite rearing followed the methods used in our recent work [26] composed mostly of a commercial pet food mixture. *Necrobia rufipes* were reared as described by Hasan and Phillips [6] on a 1:1:1 ratio of dried fish, ham pieces, and whole and ground commercial pet food consisting of a minimum of 23% crude protein, 12% crude fat, and 4% crude fiber at a 14% moisture content. Adult *N. rufipes* were placed in a ventilated 850 mL glass rearing jar, there were 20–50 beetles with approximately 50 g of diet, and incubated in a cabinet held at 27.5 ± 1.0 °C and 70.0 ± 2.0% relative humidity (R.H.) in darkness.

### 2.2. Mite Life Stage Separation

Healthy groups of mixed protonymphs, tritonymphs, and adults were classified as mobile mites for this research, and the largest mites were selected from the laboratory colonies using a one-bristled Talkon^®^ bristle paintbrush. For fumigation trials, twenty mobile-stage mites were placed into 1.8 mL glass shell vials (Thermo Fisher Scientific, Waltham, MA, USA) that contained a 27 mm^3^ piece of country ham. The vials were labeled and covered with 30 μm of mesh screening that was held in place with a ventilated plastic stopper. Vials were stored at 22.5 ± 2.5 °C and 70.0 ± 2.0% R.H. for up to 24 h prior to fumigation.

Mite eggs for testing were generated by first removing 50 healthy adult mites, mainly gravid females, from the filter paper lids of active mite cultures. These adults were placed into a 118 mL glass jar with 7.5 g of cooked mite diet mixture and covered with a labeled P5 Fisherbrand 7.0 cm diameter filter paper and a metal ring. The adults were left for 48 h in a double water bath of an ambient temperature at 25 °C and 70% R.H. in total darkness. The mite eggs were then individually removed using a one bristled Talkon^®^ bristle paintbrush, and 20 eggs were placed on a piece of double-sided sticky cellophane tape that was attached to a piece of black construction paper onto which a 27 mm^3^ piece of ham was also placed. The egg sheets of 20 eggs with ham were placed into 1.8 mL shell vials and covered with a ventilated lid as described above. Vials were stored at 22.5 ± 2.5 °C and 70.0 ± 2.0% R.H. for up to 3 h before fumigation.

### 2.3. Efficacy Bioassays

#### 2.3.1. Fumigant Concentration Mortality Assays

Propylene oxide and ethyl formate were purchased from Sigma Aldrich (Milwaukee, WI, USA) at purities of ≥99 and 97%, respectively. One-liter glass wide-mouthed jars served as fumigation chambers and were loaded with 1.8 mL vials of 27 mm^3^ pieces of 6-month-aged country ham (Tripp Country Hams, Brownsville, TN, USA). Each chamber contained 2 vials, each with 20 mobile mites and 2 other vials, each with 10 eggs. Each jar had an 11.0 mL glass vial with 2 mL of water to provide approximately 70% RH. A single fumigation chamber represented one experimental unit for exposure to a targeted gas concentration. Precise volumes of liquid fumigants, which were chilled to prevent evaporation while pipetting, were applied to each jar with calibrated micropipettes with tips that were either 0.5–10 μL (Molecular Technologies, AlphaαPette), 2–20 μL, or 20–200 μL (Rainin, Classic) in volume. The target concentrations for each jar were calculated as mg/L based on the volume of the jar that was used and the density of the liquid fumigant as provided by the manufacturer, which was 0.859 g/cm^3^ for PPO and 0.917 g/cm^3^ for EF. Liquids were applied along the internal sidewall of each glass jar inside a fume hood and the jars were immediately sealed and secured with a gas-tight metal canning lid and screw ring. The jars were placed in an incubator set at 25.0 ± 1.0 °C with 70 ± 2.0% R.H. and a 16 h photoperiod for 24 h. After 24 h, the jars were opened and ventilated in the fume hood for 10 min. The vials were then removed from fumigation jars and placed into a desiccator chamber with a saturated NaCl solution at room temperature 22.5 ± 2.5 °C and 70.0 ± 2.0% R.H. for a recovery period of 72 h for the mobiles and 168 h for the eggs. The mortality of the mobiles was determined by the inability to move, and the eggs were scored as dead by failing to hatch to larvae. Any mobile stage that did not physically react following a 30 s viewing at the end of the recovery period was scored as dead. Fumigant concentration–response assays were replicated two to six times for each concentration. Limited numbers of *N. rufipes* life stages prevented concentration–mortality studies. Mixed life stage fumigations of *N rufipes* with PPO and EF at high doses are described below.

#### 2.3.2. Time–Mortality Assays

**Figure 1:**
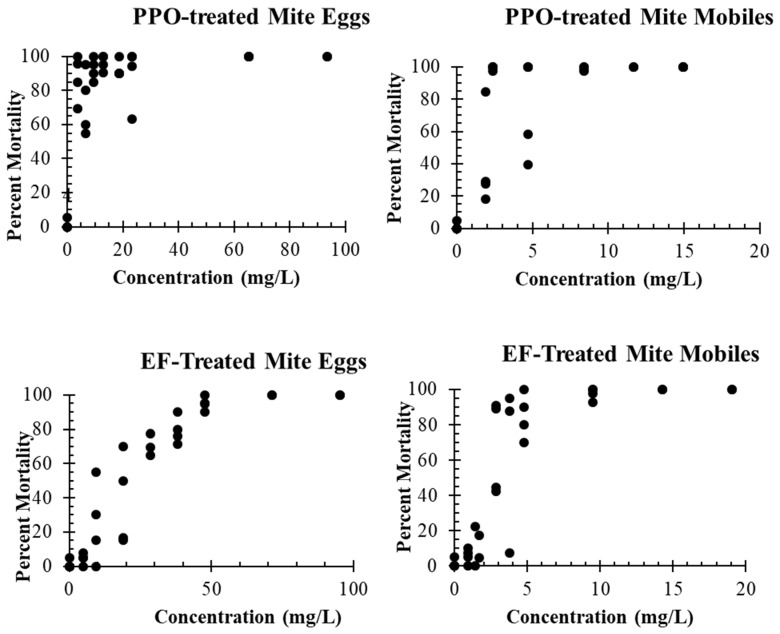
Mortality of *T. putrescentiae* eggs (**left**) and combined mobile stages (**right**) exposed to a range of concentrations of either propylene oxide (PPO, top two graphs) or ethyl formate (EF, bottom two graphs) for 24 h at 25 °C. Each point represents the percentage killed of either 40 mobile mites or 20 mite eggs in a single fumigation jar. Four jars were fumigated at most gas concentrations.

Time–mortality assays with *T. putrescentiae* were conducted with the same methods described above for the concentration–mortality assays. Eggs were used for the exposure time experiments as the initial experiments on concentration–mortality assays (see Figure 1) determine eggs to be the most tolerant life stage to both fumigants. The gas concentrations for the exposure time fumigations were 71.42 mg/L of PPO and 70.09 mg/L of EF, as these concentrations caused 100% mortality to eggs in the first experiments (see Figure 1). We tested fumigant exposure times of 1, 2, 3, 4, 5, 6, 9, and 12 h for each gas and there were two replicates (fumigation chambers) for each exposure time.

#### 2.3.3. Mixed Life Stage Fumigation

The ability of PPO and EF to kill lab populations of *T. putrescentiae* and *N. rufipes* was evaluated in mixed life stage fumigations. Fumigations of large numbers of mites were conducted in ventilated 118 mL glass jars. Food for these was 25 g of a pet-food-based rearing diet developed earlier in our lab [27]. A Talkon^®^ bristle paintbrush was used to gently brush the underside of the mite culture’s filter paper lid into a tared 11 mL glass vial until 55 mg of mobile mites and eggs (~7000 individuals) was accumulated in each opened vial. The mites in the vial were then carefully placed onto the food in each 118 mL jar, and the jars were secured with a piece of 30 µm mesh (Fisher Scientific) screening over the opening which was fitted tightly to the jar with a screw-on metal lid ring. Mixed life stage mite jars were then placed into a soapy water bath and held for up to 12 h at 22.5 ± 2.5 °C and 70.0 ± 2.0% R.H. to provide time for the mites to acclimate and continue oviposition.

The toxicity of the liquid fumigants on the mixed life stages of *N. rufipes* was evaluated using methods similar to those of Sağlam et al. [28]. Twenty-six adults from 6-week-old lab cultures were isolated using vacuum tweezers and a U.S. No. 20 sieve into a 118 mL glass jar that contained 7 g of fish meal that had been sieved with a U.S. 40 sieve, 7 g of ground pet food sieved with a U.S. 40 sieve, and 7 g of whole pet food kibbles to create mixed life stage cultures. These cultures were then placed into an incubator set at 29.0 ± 1.0 °C with 70.0 ± 2.0% R.H. under 16:8 L:D. An additional 26 adults were added to each culture 7 days later to create overlapping generations of immatures. All adults were removed with vacuum tweezers 35 d from the original infestation date, and colonies were left to continue developing with the same incubator settings for a total of 75 d, which contained new adults and many ages of all immatures.

The mixed life stage jars of *T. putrescentiae* or *N. rufipes* were placed into 1 L wide-mouthed glass jars containing a 11 mL vial with 2 mL of water for humidity. Fumigant concentrations at 1× and 2× the concentration were used that consistently gave 100% mortality of *T. putrescentiae* eggs (the most tolerant life stage) in the initial fumigant concentration–mortality studies (Figure 1 and Table 1). The fumigation jars were secured as before and placed in an incubator set at 25.0 ± 1.0 °C with 70.0 ± 2.0% R.H. and a 16 h photoperiod for 24 h, after which the jars were vented in the fume hood for 10 min. Mixed life stage colonies of *T. putrescentiae* were removed from their fumigation jars and placed into a humidified glass desiccator at 70.0 ± 2.0% R.H. at 22.5 ± 2.5 °C for a recovery period of 72 h. Mite jars were then placed into a 30 ± 2.0 °C water bath, and a 5 × 1 cm piece of paper was held on the food bait in each jar to remove the mobile mites for counting. The mixed life stage jars were placed back into the desiccator under previously described conditions for another four days for a total of seven days post-treatment, after which all mobile mites in each jar were counted. These represent any mobile mites that survived fumigation or new mobile mites that hatched from eggs that survived fumigation. *T. putrescentiae* mixed life stage fumigations were replicated five times. The mixed life stage jars of *N. rufipes* were removed from 24 h fumigated jars, following 10 min aeration, and were placed into an incubator at 29.0 ± 1.0 °C with 70.0 ± 2.0% R.H. 16:8 L:D for a recovery period of 72 h, after which any surviving adults were counted. The beetle jars were then placed back into the same incubator and newly emerged adult beetles were removed and counted every 14 d up to a total of 84 d post-fumigation. Any living adult beetles removed and counted over those 84 days resulted from immature beetles that survived fumigation. *Necrobia rufipes* mixed life stage fumigations were replicated four times.

### 2.4. Sorption and Desorption Bioassays

The term “sorption” in fumigation refers to a decrease in gas concentration not resulting from a leak in the structure being fumigated. The desorption of a fumigant refers to the release of a fumigant from a certain commodity at some time after the ventilation of the structure or chamber that was fumigated [20]. The qualitative detection of the sorption and desorption of EF or PPO related to the commodity was determined with a variation in the mortality of *T. putrescentiae* caused by the presence of a commodity in the fumigation chamber compared to the mortality of the mites’ empty chamber which was fumigated in the same way but with no commodity. Sorption bioassays were conducted in 2 L wide-mouthed glass jars loaded with 200 g of a given commodity: either a piece of country ham, commercial dog food kibbles (Beneful dry dog food, Nestle-Purina, Neenah, WI, USA), or organic fish meal (PowerGrow Systems, Vineyard, UT, USA). Two small 1.8 mL ventilated glass vials, each with 20 eggs (the most tolerant life stage), a 5 cm diameter glass Petri dish, and a glass vial containing 4 mL of water for humidity, were placed in all the jars. The glass vials and the Petri dish were placed resting on the commodity. PPO was added at 93.5 mg/L, and EF was added at 95.2 mg/L to the 3 cm diameter glass Petri dish for evaporation to prevent the liquids from contacting the commodity. The 2 L jars were immediately sealed and secured with a metal gas-tight lid and placed in an incubator set at 25.0 ± 1.0 °C with 70.0 ± 1.0% R.H. in 16:8 (L:D) for either 12 or 24 h, after which the jars were ventilated for 10 min. The vials of eggs were then removed from the fumigation jars and placed into a desiccator at 29.0 ± 1.0 °C with 70.0 ± 2.0% R.H. and 16:8 L:D for a 72 h recovery period for mobile mites and were subsequently checked at 168 h for the mobiles hatched from fumigated eggs.

After the 10 min ventilation of fumigation jars described above, desorption bioassays were conducted in the same fumigation jars containing the fumigated commodity by adding two 1.8 mL vials each containing a fresh untreated 27 mm^3^ piece of ham and 20 mite eggs in a vial secured with a ventilated lid with 30 µm mesh screening. We therefore evaluated the presence of gas desorbed from the commodity by using an egg toxicity assay. The 2 L jars were sealed and secured with a gas-tight lid and were placed in an incubator set at 25.0 ± 1.0 °C with 70.0 ± 1.0% R.H. 16 h photoperiod for 12 h and 24 h. Mortality was accessed by the failure of an egg to hatch after a three-day recovery period. Sorption and desorption bioassays were replicated four times.

### 2.5. Data Analyses

Fumigant concentration mortality assays and time response assays were analyzed using probit regression SAS Studio^®^ 5.1 software [29]. Sorption and desorption mortality assay data were transformed with an angular transformation and tested for normality and individually analyzed using a one-way ANOVA with general linear mixed models (GLMMIX) [29]. Means were separated using Tukey’s HSD (honestly significant difference) post hoc test at *p* < 0.05.

## 3. Results

### 3.1. Fumigant Concentration Mortality Assays

The mobile life stages of *T. putrescentieae* were very susceptible to both PPO and EF to the point that low concentrations of either gas at 9, 12, and 15 mg/L gave 100% mortality in all trials in Figure 1. These results for mobile life stages could not be placed into a probit analysis for a comparison to egg mortality, but the low tolerance of the mobile stages was very clear. Concentration–mortality assays with eggs clearly showed that the eggs were much more tolerant than mobile stages for mortality from both gases. Probit analyses for the mortality of *T. putrescentiae* eggs exposed to propylene oxide and ethyl formate for 24 h are reported in Table 1. The Pearson goodness-of-fit (χ^2^) test showed that the probit regression model’s fit to the observed data was significant (*p* < 0.05) for both EF and PPO. Since the *p*-values for the tests were low, variances and covariances for each test were adjusted by a heterogeneity factor (Chi-square value (χ^2^) divided by the degrees of freedom (df)), and the critical value from the t-distribution was then used to compute the fiducial limits for the LC_50_ and LC_99_ [29]. Fiducial limits for the predicted concentrations for mortality were not available from our computation due to the wide variation in the concentrations, yielding high and low levels of mortality.

### 3.2. Time–Mortality Results

Results of the time–mortality experiments for *T. putrescentiae* are reported in Table 2. PPO could elicit only 56.6 ± 6.6% mortality of *T. putrescentiae* eggs within a 12 h exposure at a concentration of 71.42 mg/L. However, EF achieved 100 ± 0.0% egg mortality at 6 h and longer exposures using a concentration of 70.09 mg/L.

### 3.3. Mixed Life Stage Fumigation Results

The results of the mixed life stage fumigations are reported in Table 3. *T. putrescentiae* averaged less than three mobile mites surviving a PPO application at 93.5 mg/L and less than one mobile to survive at an application rate of 186.9 mg/L. The fumigation of mite mixed life stage cultures using EF at 95.2 mg/L resulted in mobile mite survival of less than one mite on average and at 190.4 mg/L, all the mites were dead, and no mobile stages were observed at seven days after treatment. *Necrobia rufipes* averaged about 25 adults emerging from untreated mixed life stage cultures after 42 d. For fumigated *N. rufipes* mixed life stage cultures, we counted an average of three beetles emerging from cultures treated with PPO at 93.5 mg/L and EF at 95.2 mg/L after 84 days. Beetles were fully controlled when fumigated with PPO at 186.9 mg/L and with EF at 190.4 mg/L.

### 3.4. Sorption and Desorption Bioassay Results

Sorption, if it occurred, had essentially no effect on the mortality of the mite eggs fumigated with either gas. No eggs hatched in empty jars fumigated with either PPO or EF for either 12 h or 24 h. Survival was not significantly increased by any sorption of PPO during the 24 h exposure times, with only 1 or 2 eggs hatching out of the 80 which were treated (F = 1.10, df = 3, *p* = 0.3881). For EF, however, 5% of treated eggs hatched when fumigated with EF for 12 h in the presence of fish meal compared to jars without commodity (F = 52.39, df = 3, *p* < 0.0001) and 0.1% hatched after 24 h of fumigation with fish meal (F = 83.65, df = 3, *p* < 0.0001). The desorption of either gas following a 10 min. ventilation of the fumigated jars did not cause any mortality, as per failure to hatch, of the newly placed eggs that was different from the levels of non-hatch in the ventilated empty jar with no commodity.

## 4. Discussion

The results from the fumigation experiments with PPO and EF found that the storage pests *Tyrophagus putrescentiae* and *Necrobia rufipes* could be controlled at reasonable gas concentrations and at typical room temperature in a 24 h exposure. The eggs of *T. putrescentiae* were the most tolerant life stage for the toxicity of both fumigants as the gas levels needed for the 100% kill of eggs were seven-fold higher than the concentrations needed to control a mixture of mobile mite stages. Large mixed life stage cultures of both species were fumigated at high concentrations, and no mature mites or adult beetles survived. If any successful registrants were to recommend the application of either PPO or EF at the effective concentrations we report here, of about 190 mg/L (Table 3), then concerns about flammability and effective application rates would need to be addressed. Both EF and PPO are highly flammable [14,17] and require the addition of a fire retardant such as carbon dioxide at high levels, as with the formulations already in use e.g., [16,30]. Additionally, as discussed below, sorption may be very significant for other commodities [31] in commercial-scale storage structures being treated that the treatment concentration would need to be much higher than 190 mg/L to provide enough gas for effective control.

Sorption, if any, from the presence of either dried fish meal, aged ham, or kibbled pet food in the fumigation chamber did not affect the toxicity of PPO or EF to mite eggs. Desorption bioassays following the ventilation and resealing of fumigation jars with any commodity tested had no significant effect on egg hatch. These results suggest that commonly mite-infested commodities of ham, fish meal, and pet food may result in very little to no sorption of EF or PPO that could negatively affect the successful fumigation of *T. putrescentiae* or result in the release of toxic gas after the ventilation of a fumigated building. Interestingly, our sorption results with ham, dog food, and fish meal differ greatly from our earlier work in which there was 53–94% loss of these two gases in the presence of the common field crops dried wheat, wheat flour, corn, and beans [31]. In that study, we fumigated laboratory chambers with a large percentage of the volume filled with commodity for sorption studies. The studies we report here used very small amounts of commodity in the jars, and thus may not have represented aa typical industrial fumigation of stored commodity. Future work on the sorption of EF and PPO should be pursued in the future using analytical chemistry methods to measure gas concentrations, and test other commodities taking up a large proportion of space in the fumigation chambers.

We found that *T. putrescentiae* eggs were the most tolerant life stage for both EF and PPO. Our earlier work also showed the tolerance of *T. putrescentiae* eggs for several other fumigants used on stored products. *Tyrophagus putrescentiae* fumigated with either hydrogen phosphide (phosphine) or methyl bromide required slightly higher concentrations of each to kill eggs compared to the concentrations needed for mobile stages. Hasan et al., in 2019, reported that the LC50 concentration of phosphine to kill *T. putresentiae* eggs was 1.58-fold more than that needed to kill mobiles, and 1.13-fold higher for methyl bromide to kill eggs relative to that needed to kill mobiles. Hasan et al., in 2021, reported that LC50s which showed eggs of *N. rufipes* were more tolerant to the fumigant sulfuryl fluoride than were larvae by 5.31-fold and 11.64-fold more than that needed for adults [12]. The same study on sulfuryl fluoride which reported the tolerance of *T. putrescentiae* eggs based on LC50 was 2.46-fold higher than for mixed mobile stages. However, regarding controlled atmospheres of either high-carbon-dioxide or low-oxygen treatments [32], *N. rufipes* larvae and pupae were more tolerant to high CO_2_ concentrations than the eggs, and the mobile mites were marginally more tolerant to CO_2_ that mite eggs. The same study reported that *N. rufipes* larvae exposed to low-oxygen atmospheres at 25 mm atm. were more tolerant than eggs, while for mites, the eggs, as with synthetic fumigants, were more tolerant than mobiles to low oxygen by a 2.9-fold margin. As with any fumigation of pests at a commercial level, the required fumigant concentration and/or hold time should be determined by conditions for the best kill to the most tolerant life stage.

A broad objective for the work reported here was to provide information on fumigants that may serve as alternatives to methyl bromide for use on dry-cured hams, and durable stored food products in general, that are both safe and effective. Both PPO and EF have been tested and found effective for many pest insect species associated with various stored products (see reviews by Jimenez et al. [33] and Ryan and Da Lima [34]). Both compounds are considered safe in food products at certain levels, and thus may have potential for registration to control pests on dried hams and other durable foods [16,35,36]. Propylene oxide is currently registered by the USA Environmental Protection Agency [35] as a fumigant and sterilization agent for certain storage insects and bacteria that may be infesting herbs, spices, cacao bean, tree nuts, dried figs, and raisins. As for food safety, PPO is registered as a food additive by the US Food and Drug Administration for use as an antimicrobial agent [16]. EF is not registered as a fumigant in the USA, but it is among a special group of food additives in the USA designated as generally regarded as safe (GRAS) [36]. EF is commercially available under the product name VAPORMATE™ [30], as a stored fumigant in Australia but not in the USA.

This study shows that the simple gases evaporated from liquid PPO and EF are effective at controlling *T. putrescentiae* and *N. rufipes*, and therefore represent viable alternatives to methyl bromide for the management of these pests of dry-cured hams. Although we did not quantitatively measure PPO or EF sorption or residues in the fish meal, ham, or pet food studies, our results show that different commodities can have different sorption qualities, which can impact pest mortality. Earlier work showed PPO to have high sorption rates in several commodities [20,23,37]. EF has also been reported to have sorption properties in wheat [38]. The build-up of PPO has still been reported to be toxic enough to achieve the mortality of *Plodia interpunctella* (Hübner) in peanuts and walnuts [23], and now a small number of *T. putrescentiae* have been killed in our work due to desorption. Future studies should be conducted using commercially formulated PPO and EF in commercial-scale or near-commercial-scale structures containing hams and perhaps other products artificially infested with *T. putrescentiae* and other ham pests. Such future studies could evaluate the occurrence of leakage, sorption, residues, temperature, and variable concentrations. Furthermore, more information could help support regulatory approval from government agencies as pesticides for use on more commodities in many countries.

## Figures and Tables

**Table 1:** Probit analysis of mortality response for *T. putrescentiae* eggs to concentrations of either propylene oxide (PPO) or ethyl formate (EF) following a 24 h exposure at 25° C.

	n	Slope ± SE	Intercept ± SE	LC_50_ (F.L.) (mg/L)	LC_99_ (F.L.) (mg/L)	χ^2^	DF	*p* > χ^2^	R^2^
PPO	416	1.28 ± 0.63	0.08 ± 0.57	0.87 (N.A.)	56.48 (N.A.)	54.48	18	<0.01	0.16
EF	1647	0.98 ± 0.082	−0.74 ± 0.11	19.74 (17.90–21.52)	96.17 (74.16–141.90)	210.69	31	<0.01	0.19

If *p* > χ^2^ is larger than 0.05, then this indicates a significant difference between the observed data and the expected values regression line. F.L. = fiducial level. N.A. indicates that the values for fiducial levels are “Not Available” due to the lack of fit of the raw data to the probit regression line so that the FLs would be so high and not useful to describe the variation around the calculated LC value.

**Table 2:** Average mortality response and standard error of *T. putrescentiae* at various exposure times to PPO and EF at 25 °C.

	Average Percent Mortality (S.E.)	
Time (h)	PPO Treated Eggs at 71.42 mg/L	EF Treated Eggs at 70.09 mg/L
0	1.2 (±1.2)	0.3 (±0.3)
1	5.3 (±5.3)	25.0 (±10.0)
2	2.3 (±2.2)	77.5 (±7.5)
3	13.1 (±3.6)	95.0 (±0.0)
4	4.8 (±4.8)	94.7 (±5.3)
5	19.3 (±3.5)	95.2 (±4.8)
6	31.5 (±8.5)	100.0 (±0.0)
9	51.9 (±6.9)	100.0 (±0.0)
12	56.6 (±6.6)	100.0 (±0.0)

**Table 3:** The average and standard error of emerged adults from treated mixed life stage jars in days after fumigation of PPO and EF on *Tyrophagus putrescentiae* (T. p.) and *Necrobia rufipes* (N. r.) mixed life stage fumigations at concentrations 1× and 2× the concentrations that cause 100% mortality. NA refers to data not available from untreated jars of *Necrobia rufipes* in the EF experiment.

		Mean ± SE (n = 5) Emergence at Different Gas Concentrations (mg/L)					
		PPO			EF		
Species Tested	Recovery Days	0 mg/L	93.5 mg/L	186.9 mg/L	0 mg/L	95.2 mg/L	190.4 mg/L
T. p.	3	4431.5 ± 1292.5	2.4 ± 1.0	0.4 ± 0.4	7884.5 ± 587.5	0.0 ± 0.0	0.0 ± 0.0
	7	18,704.5 ± 442.5	0.6 ± 0.4	0.0 ± 0.0	15,593.5 ± 1491.5	0.6 ± 0.4	0.0 ± 0.0
							
N. r.	3	12.8 ± 2.3	0.0 ± 0.0	0.0 ± 0.0	10.0 ± 1.1	0.0 ± 0.0	0.0 ± 0.0
	14	4.0 ± 0.7	1.5 ± 0.6	0.0 ± 0.0	NA	1.5 ± 0.5	0.0 ± 0.0
	28	5.8 ± 1.1	1.8 ± 1.8	0.0 ± 0.0	NA	1.0 ± 0.4	0.0 ± 0.0
	42	2.8 ± 1.3	1.3 ± 1.3	0.0 ± 0.0	NA	0.0 ± 0.0	0.0 ± 0.0
	56	0.0 ± 0.0	0.0 ± 0.0	0.0 ± 0.0	NA	0.0 ± 0.0	0.0 ± 0.0
	70	4.0 ± 0.7	0.8 ± 0.8	0.0 ± 0.0	NA	0.5 ± 0.3	0.0 ± 0.0
	84	3.8 ± 0.9	0.0 ± 0.0	0.0 ± 0.0	NA	0.0 ± 0.0	0.0 ± 0.0

## Data Availability

The data presented in this study are available on request from the corresponding author. The data are not publicly available due to privacy restrictions on the authors.
